# GOT1/AST1 expression status as a prognostic biomarker in pancreatic ductal adenocarcinoma

**DOI:** 10.18632/oncotarget.2799

**Published:** 2015-02-19

**Authors:** Fenja M Feld, Philipp D Nagel, Stephanie E Weissinger, Claudia Welke, Albrecht Stenzinger, Peter Möller, Jochen K Lennerz

**Affiliations:** ^1^ Institute of Pathology, University Ulm 89081, Germany; ^2^ Comprehensive Cancer Center Ulm, University Hospital Ulm 89081, Germany; ^3^ University Hospital Heidelberg, Department of Pathology, University Heidelberg 69120, Germany

**Keywords:** pancreatic cancer, glutamic oxaloacetatic transaminase 1, *KRAS*, PDAC, biomarker

## Abstract

Prognostication in pancreatic ductal adenocarcinoma (PDAC) remains a challenge. Recently, a link between mutated *KRAS* and glutamic-oxaloacetic transaminase (GOT1/AST1) has been described as part of the metabolic reprogramming in PDAC. The clinical relevance of this novel metabolic KRAS-GOT1 link has not been determined in primary human patient samples. Here we studied the GOT1 expression status as a prognostic biomarker in PDAC. We employed three independent PDAC cohorts with clinicopathological- and follow-up data: a) ICGC, comprising 57 patients with whole-exome sequencing and genome-wide expression profiling; b) ULM, composed of 122 surgically-treated patients with tissue-samples and *KRAS* status; c) a *validation cohort* of 140 primary diagnostic biopsy samples. GOT1 expression was assessed by RNA level (ICGC) or immunolabeling (ULM/validation cohort). GOT1 expression varied (ICGC) and correlation with the *KRAS* mutation- and expression status was imperfect (*P* = 0.2, ICGC; *P* = 0.8, ULM). Clinicopathological characteristics did not differ when patients were separated based on GOT1 high vs. low (*P* = 0.08–1.0); however, overall survival was longer in patients with GOT1-expressing tumors (*P* = 0.093, ICGC; *P* = 0.049, ULM). Multivariate analysis confirmed GOT1 as an independent prognostic marker (*P* = 0.009). Assessment in univariate (*P* = 0.002) and multivariate models in the *validation cohort* (*P* = 0.019), containing 66% stage IV patients, confirmed the independency of GOT1.

We propose the GOT1 expression status as a simple and reliable prognostic biomarker in pancreatic ductal adenocarcinoma.

## INTRODUCTION

Mortality rates in pancreatic ductal adenocarcinoma (PDAC) have not changed in decades and it remains the fourth leading cause of cancer related deaths [1, 2], with an overall 5-year survival rate of < 5% [1, 3] and a median overall survival time of 3–12 months [3–5]. Therefore, tremendous efforts are being put into assessing novel therapies in pancreatic cancer [4–10]. In parallel, delineation of the underlying molecular-genetic aberrations is under way (e.g., genome-wide mutational landscapes; expression profiling studies; etc.) [5, 11–15]; however, recognizing the aggressive nature of this cancer type, the treating oncologist is in need of robust prognostic biomarkers [11, 16–19]. The current WHO-classification brings it to the point: “None of the many molecular prognostic indicators reported has yet become established in routine clinical practice” [20].

**Figure 1:**
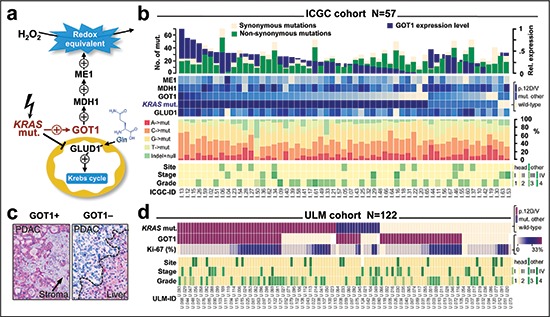
Overview of the recently described *KRAS* function in pancreatic ductal adenocarcinoma and findings in screening cohorts **(a)** Mutant *KRAS* increases redox-equivalents at cost of energy. Reprogramming includes the induction of GOT1, which is essential for pancreatic cancer cell survival; modeled after *Son et al., 2013*. **(b)** *Findings in the ICGC cohort*. Panel allows comparison of sample-specific mutations (as determined by whole-exome sequencing; upper histogram), *KRAS* mutation status and enzyme expression levels (upper heatmap), relative fraction of mutations (column graphs), and key clinicopathological features of samples at the case-level (columns). There is no correlation between *KRAS* mutations and GOT1 expression level (details see results). **(c)** *Immunohistochemical staining pattern of GOT1* in a positive (left) and a negative case (right). Positivity is defined as cytoplasmic GOT1 immunoreactivity (red) that is more intense than the stroma (arrow). Note, liver cells show immunoreactivity and act as an internal staining control. **(d)** *Findings in the ULM cohort*. Panel compares *KRAS* mutation status (by pyrosequencing), GOT1 protein expression, proliferation ratio (Ki-67), and key clinicopathological features at the case-level (columns). There is also no correlation between *KRAS* mutation status and GOT1 protein expression. **Abbreviations:** GOT1, glutamic oxaloacetic transaminase 1; GLUD1, glutamate dehydrogenase 1; MDH1, malate dehydrogenase1; ME1, malic enzyme 1.

Recently, a novel metabolic function of the highly prevalent *KRAS* mutation in PDAC has been identified [21]. Briefly, pancreatic cancer cells derive their energy in large parts from glutamine, which serves as an indirect substrate for the Krebs cycle and renders the cancer cell dependent on glutamine [21, 22]. This glutamine addiction [23] is striking because glutamine is a nonessential amino acid that can be synthesized from glucose [22]. Son et al., have now shown that the *KRAS* mutation modulates the associated metabolic pathways by inducing GOT1 (in short, *KRAS*-GOT1 link) [21, 24]. The resulting upregulation shifts glutamine towards enzymes that orchestrate cell growth and the redox maintenance system (Figure 1a). Thereby, one hallmark mutation of pancreatic carcinoma, *KRAS*, mediates a shift in the cancer cell's glutamine-based energy supply system towards other pathways. Currently, the clinical relevance of this novel metabolic *KRAS*-GOT1 link has not been determined in primary human patient samples.

Here, we screened a genome-wide dataset of human PDAC for evidence of the *KRAS*-GOT1 link and performed a biomarker study assessing the clinical and prognostic relevance of GOT1. Our findings identify GOT1 expression as an independent prognostic biomarker in PDAC.

## RESULTS

### The KRAS-GOT1 link is not uniformly present in pancreatic ductal adenocarcinoma samples

Since the clinical relevance of the recently described *KRAS*-GOT1 link [21] is currently unknown in primary human patient samples, we examined the freely available ICGC-PDAC dataset. An overview of the patient cohort with available exome sequencing information [25] and gene expression profiling [26], is provided in Table 1 and Figure 1b. First, we checked for correlation of *KRAS* to enzyme mRNA expression levels; however, found no correlation with the two directly linked enzymes GLUD1 and GOT1 (*P*-range: 0.2–0.8; Supplemental Figure 1b/c). This was also the case when restricting the analysis to mutant *KRAS* or those mutations for which the link has been described (p.G12D/V; not shown) [21]. Due to a relatively higher clonogenic potential and more pronounced growth dependence of PDAC cell lines [21], we subsequently focused on GOT1. Interestingly, tumors differed by GOT1 expression levels and we separated patients with GOT1-high vs. –low tumors (Supplemental Figure 1a). As one of the key redox mediators in PDAC, GOT1 is directly involved in the oxygen-radical salvage and we examined DNA-base exchange patterns, number of synonymous or non-synonymous mutations as well as number of recurrent genomic mutations; however, we did not find significant differences between GOT1-high and –low tumors (Figure 1b, Supplemental Figure 1d and 2). Since the *KRAS*-GOT1 link has been described at the RNA level [21], the ICGC dataset with expression levels appears adequate; yet, proteins are the mediators of biological function. Thus, we also established GOT1-immunolabeling (Figure 1c and Supplemental Figure 3) and tested our *KRAS*-genotyped PDAC cohort (*ULM*, Figure 1, Table 1) [27]. Immunolabeling showed that 65% of cases (n = 77/118) were GOT1 positive; however, only about one-third of all cases were *KRAS* mutated *and* GOT1 positive (n = 46/118). Thus, probing for the *KRAS*-GOT1 link at the RNA and protein level in two cohorts demonstrated that GOT1 is apparently not uniformly expressed; at least not in resected PDAC samples.

**Table 1:** Clinicopathological characteristics of the ICGC- and ULM cohorts

Characteristic	ICGC N = 57	ULM N = 122	*P*
**Male sex, *n* (%)**	34 (60%)	71 (58%)	0.87
**Age – yr**			
Median	66.0	66.56	
Range	34–87	40–82	
Age ≥ 65 years	29 (51%)	69 (57%)	0.52
**Site**			
Head	47 (82%)	105 (87%)	0.50
Body/Tail	10 (18%)	16 (14%)	
**Tumor Size, *n* (%)**			
T1/2	9 (16%)	13 (11%)	0.34
T3/4	48 (84%)	109 (89%)	
**Nodal Metastasis, *n* (%)**			
N0	11 (20%)	39 (32%)	0.11
N1	45 (80%)	83 (68%)	
**Systemic Metastasis, *n* (%)**			
M0	53 (93%)	113 (93%)	1.00
M1	4 (7%)	9 (7%)	
**Stage Grouping, *n* (%)**			
I/II	53 (93%)	107 (88%)	0.44
III/IV	4 (7%)	15 (12%)	
**Histological Grade, *n* (%)**			
G1/2	34 (60%)	86 (70%)	0.17
G3/4	23 (40%)	36 (30%)	
**Surgical margins, *n* (%)**			
R0	–	92 (76%)	
R1	–	29 (24%)	

*P*-values from *t*-test or Fisher's exact test.

### Phenotype screening reveals GOT1 as an independent prognostic biomarker in pancreatic ductal adenocarcinoma

**Figure 2:**
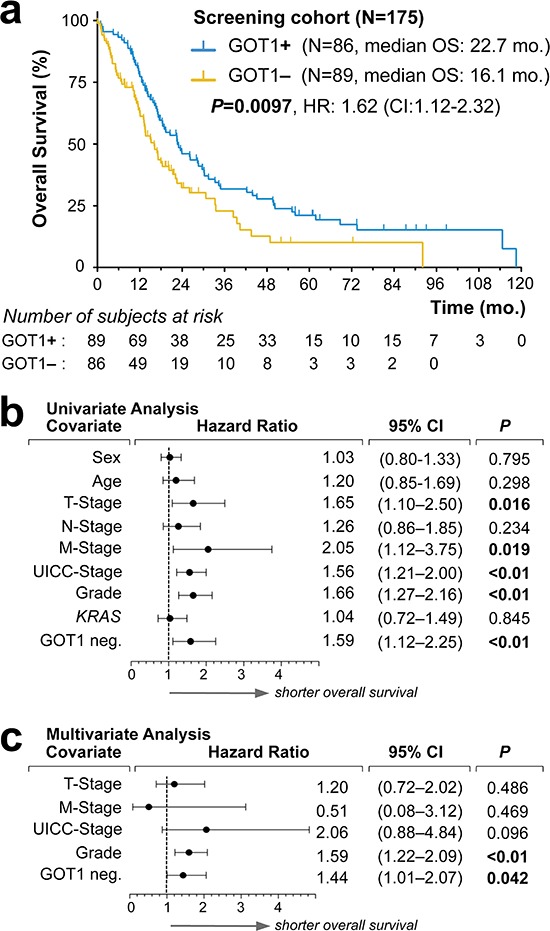
Screening of GOT1 expression status as an independent prognostic biomarker in pancreatic ductal adenocarcinoma **(a)** Kaplan-Meier estimates of outcome for GOT1 positive vs. negative samples in our screening cohort. **(b, c)**. Forest plots of log hazard ratios (HR) from univariate **(b)** and multivariate **(c)** Cox proportional regression models for overall survival according to baseline clinical characteristics. Note, for comparison of effect size among characteristics associated with shorter overall survival, we plotted GOT1-negativity (GIT1 neg.). **Abbreviations:** *KRAS*, indicates presence of mutation; mo., months; HR, hazard ratio; CI, confidence interval.

Comparison of the clinical features between the GOT1 subgroups in the ICGC dataset showed no striking differences (Figure 1b; Supplemental Table 1); however there was a trend that patients with GOT1-high tumors survived ~2 months longer (14.6 vs. 12.1 months; *P* = 0.093; Supplemental Figure 4). We followed this lead and performed immunolabeling of GOT1 in the ULM cohort (Supplemental Fig.3). For clinical usage, a positive tumor was defined as cytoplasmic staining stronger than that of stromal cells. In 4 of 122 cases, staining was intermediate to weak, and these cases were counted as positive as well. We found that overall survival of patients with GOT1-positive vs. –negative tumors differed by over 6 months and reached statistical significance (22.53 vs.15.0 months; *P* = 0.049; Supplemental Figure 4). We also compared the *KRAS*-GOT1 ‘linked’ group of patients vs. ‘other’; however, failed to find significant differences (*P* = 0.55; Supplemental Figure 4). Comparison of the clinicopathological features between the ICGC and ULM *cohort* yielded no significant differences (Table 1; Supplemental Table 1). We considered the combination of both cohorts as a *screening cohort* consisting of 179 patients (Table 2). Outcome analysis showed significantly longer survival in patients with GOT1-positive tumors (22.7 vs. 16.1 months; *P* = 0.0097; Figure 2a). Notably, the clinicopatholgical features in the GOT1-subgroups cannot explain the survival difference (Table 2). Similarly, assessments of the tumor-specific proliferation index showed no significant differences in either group (Supplemental Figure 5). To describe the prognostic value of GOT1 in comparison to other existing and clinically established factors, we performed univariate analyses (Figure 2b). Examination of hazard ratios and significance levels revealed that GOT1 is in line with other traditional prognostic factors (e.g. stage, grade). Adjusting for these significant prognosticators, subsequent multivariate testing confirmed GOT1 as an independent prognostic biomarker in PDAC (Figure 2c). GOT1 is mostly known as a serological marker; however based on our additional statistical analyses, GOT1 serology (and several other clinical and serological markers) cannot function as a surrogate marker (Supplemental Table 2, Supplemental Figure 6), and we consider immunolabeling as the most efficient way to obtain the GOT1 tumor tissue status.

**Table 2:** Clinicopathological features by GOT1 status in the *screening cohort*

Characteristic	Screening cohort N = 175	GOT1+ N = 89	GOT1− N = 86	*P* high vs. low
**Sex, n (%)**				
Male	103 (59%)	46 (52%)	57 (66%)	0.07
Female	72 (41%)	43 (48%)	29 (34%)	
**Age – yr**				
Median	66.1	66	67	0.26
range	34–87	34–87	40–82	
≥ 65 y	96	49 (55%)	47 (55%)	1.00
**Site, *n* (%)**				
Head	148 (85%)	79 (89%)	69 (81%)	0.20
Body + Tail	26 (15%)	10 (11%)	16 (19%)	
**Tumor Size, *n* (%)**				
T1/2	21 (12%)	14 (16%)	7 (8%)	0.16
T3/4	154 (88%)	75 (84%)	79 (92%)	
**Nodal Metastasis, *n* (%)**				
N0	47 (27%)	24 (27%)	23 (27%)	1.0
N1	127 (73%)	65 (73%)	62 (73%)	
**Systemic Metastasis, *n* (%)**				
M0	162 (93%)	83 (93%)	79 (92%)	0.78
M1	13 (7%)	6 (7%)	7 (8%)	
**Stage Grouping, *n* (%)**				
I/II	156 (89%)	80 (90%)	76 (88%)	0.38
III/IV	19 (11%)	9 (10%)	10 (12%)	
**Histological Grade, *n* (%)**				
G1/2	116 (66%)	65 (73%)	51 (60%)	0.08
G3/4	59 (34%)	24 (27%)	34 (40%)	

*P*-values from *t*-test or Fisher's exact test.

### Validation of GOT1 as a prognostic biomarker in pancreatic ductal adenocarcinoma

**Figure 3:**
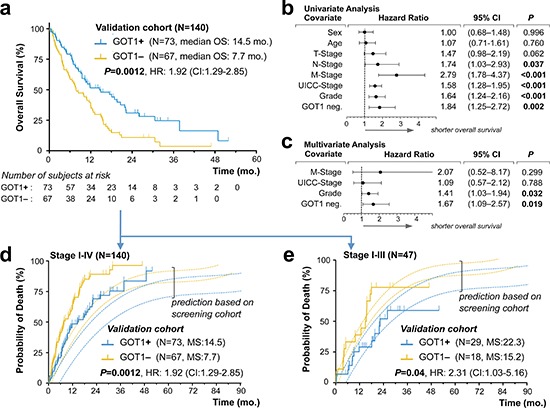
Validation of GOT1 expression status as an independent prognostic biomarker in pancreatic ductal adenocarcinoma **(a)** Kaplan–Meier estimates of outcome for GOT1 positive vs. negative samples in our *validation cohort*. **(b, c)**. Forest plots of log hazard ratios (HR) from univariate **(b)** and multivariate **(c)** Cox proportional regression models for overall survival according to baseline clinical characteristics. Note, for comparison of effect size among characteristics associated with shorter overall survival, we plotted GOT1-negativity (GOT1 neg.). **(d, e)** Stacked cumulative incidence of death in the *validation cohort*, plotted over our prediction model that was built based on survival data in our screening cohort (Figure. 2). Note, the shift between validation- and prediction curves **(d)** is due to the inclusion of 93 patients with biopsy-confirmed metastatic PDAC; curves shift when comparison is restricted to patients in stage I–III*(e)*. **Abbreviations:** mo., months; HR, hazard ratio; CI, confidence interval.

As a next necessary step, a prognostic biomarker candidate has to be validated in an independent patient cohort and we employed a separate PDAC cohort consisting of 140 consecutive biopsy samples; reflecting the typical clinical scenario, i.e., including ~63% (n = 81/140) of patients with stage IV disease at time of presentation (Table 3). In these 140 patient samples, we determined the GOT1 status by immunolabling (n = 73 GOT1+ vs. n = 67 GOT1–) and found significantly longer survival in the GOT1 positive group (14.5 vs. 7.7 months; *P* = 0.0012; Figure 3a). The striking difference of over 6 months median OS was accompanied by a trend towards lower grade tumors in the GOT1-positive subgroup and a higher rate of metastasis in the GOT1-negative subgroup (Table 3). To assess the relative effect of these factors, we performed uni- and multivariate testing and validated that GOT1 is independent from these factors and actually outperformed other prognostically relevant factors in this cohort (Figure 3b/c).

**Table 3:** Clinicopathological features by GOT1 status in the *validation cohort*

Characteristic	Validation Cohort N = 140	GOT1+ N = 73	GOT1– N = 67	*P* high vs. low
**Sex, *n* (%)**				
male	70 (50%)	32 (44%)	38 (57%)	0.18
female	70 (50%)	41 (56%)	29 (43%)	
**Age**				
Median	71	71	70	0.70
Range	21–89	21–89	33–85	
Age ≥ 65 n (%)	96 (69%)	50 (68%)	46 (69%)	1.0
**Site, *n* (%)**				
Head	80 (58%)	42 (58%)	38 (57%)	0.89
Body	37 (26%)	20 (27%)	17 (25%)	
Tail	23 (16%)	11 (15%)	12 (18%)	
**Tumor Size, *n* (%)**				
T1/2	10 (12%)	7 (14%)	3 (10%)	0.73
T3/4	70 (88%)	42 (86%)	28 (90%)	
**Nodal Metastasis, *n* (%)**				
N0	22 (28%)	15 (31%)	7 (23%)	0.45
N1	57 (72%)	33 (69%)	24 (77%)	
**Systemic Metastasis, *n* (%)**				
M0	51 (39%)	33 (49%)	18 (29%)	0.03
M1	79 (61%)	35 (51%)	44 (71%)	
Stage Grouping*, *n* (%)				
I/II	46* (36%)	29 (44%)	17 (27%)	0.07
III/IV	82* (64%)	37 (56%)	45 (73%)	
Stage Grouping*, *n* (%)				
I/II/III	47 (37%)	29 (44%)	18 (29%)	0.09
IV	81 (63%)	37 (56%)	44 (71%)	
**Histological Grade, *n* (%)**				
G1/2	84 (60%)	49 (67%)	35 (52%)	0.09
G3/4	56 (40%)	24 (33%)	32 (48%)	

**Abbreviations:** GOT1, Aspartate Aminotransferase 1; Stage grouping according to AJCC. Note: we tallied both stage groupings to illustrate that there was only one stage III patient (GOT1–) in the combined advanced or systemic group (i.e., stage III/IV) group.

Based on the findings in our screening cohort we modeled a stacked event curve and superposed events observed in our *validation cohort* (Figure 3d). While the overall survival difference of ~6 months between the GOT1 subgroups was identical, events in the *validation cohort* occurred earlier in both subgroups (shifting of curves to the left; Figure 3d). Clearly, this is due to the inclusion of ~63% of patients with systemic disease in the *validation cohort* (Table 3). When restricting the validation series to the 34% patients with stage I–III disease (n = 47/140), event curves of the *screening* and *validation cohort* were in striking alignment (Figure 3e). After confirming the robustness of GOT1 immunolabeling as a diagnostic assay in biopsy samples (Supplemental Figure 7), we now propose GOT1 as a simple, efficient and reliable, independent prognostic biomarker in PDAC.

## DISCUSSION

The key dilemma of prognostication in PDAC is that a biomarker needs to stratify patient-subgroups with an overall survival of 3–12 months. Here, we report that assessing the GOT1 status in tumor tissue samples has the potential of becoming such a biomarker. Specifically, GOT1 stratifies patients into subgroups with a median overall survival difference of ~6 months. This striking difference was significant in multivariate models and validated in an independent cohort. At this point, prediction of the disease course in PDAC relies on numerous clinical, radiological and pathological factors. For example, factors such as disease-extent, resectability, and TNM-staging are firmly established [28]; and numerous adjunct prognosticators are available (e.g., grade). Given that the diagnosis of PDAC continues to rely on histopathology, one approach for prognostication is that of tumor tissue specific biomarkers. However, despite numerous analytically well-conducted studies [16, 17, 29], none of the tissue-based biomarkers have made it into clinical practice [20]. Thus, critical assessment of our finding that GOT1 actually is a prognostic biomarker is appropriate. *First*, assessing the status of GOT1 is arguably easier than obtaining higher-dimensional data (e.g., gene-expression profiling) to stratify patients into subgroups with differences in outcome. Thus, we consider obtaining the GOT1 status as an *intermediate effort* prognostic biomarker. *Second*, obtaining the GOT1 status is possible in primary diagnostic FFPE material, even when stored for years. Specifically, our data demonstrating stability of the antigen well beyond the upper 95% confidence interval of long-term survivors provides evidence that GOT1 assessment is robust. Sharing our immunolabeling protocol that employs an established [30] and commercially available antibody should expedite implementation by other groups. *Third*, for many tissue-based prognostic biomarkers confirmation in a validation cohort is lacking. We validated our findings in an independent cohort. Ideally, such confirmation should be performed in independent laboratories. *Fourth*, the true challenge for a prognostic biomarker is verification in prospective trials. Such testing will be necessary; however, our retrospective design is not necessarily a limitation but an important starting point for prospective assessment. Yet, even prospective validation cannot overcome the practical hurdle of extrapolating from the population-level estimates to judgments for individual patients [19, 31]. Clearly, the GOT1 status in PDAC will not overcome the latter problem, and some oncologist may reject such information categorically; nevertheless, based on our study that follows the REMARK guidelines [32], we envision that the GOT1 status may serve as an adjunct tool for stratification in prospective clinical trials.

The starting point for our study was the recently described *KRAS*-GOT1 link reported by Son et al., [21]. Briefly, the *KRAS*-GOT1 link establishes that of one of *the* hallmark mutations in PDAC, *KRAS*, also orchestrates metabolic reprogramming via alteration of glutamate processing. The mutated *KRAS* mediated cytoplasmic shift via GOT1 hitchhikes downstream enzyme cascades that are critical for redox balance and cell growth [21]. While the molecular biological evidence is compelling, our results indicate that at the time of resection, only a subset of patients show evidence of the metabolic reprogramming. Given that *KRAS* mutations occur early in the multi-step progress towards invasive carcinoma, the cancer cell may have an initial interest in metabolic reprogramming for survival and mutational maintenance via prevention of catastrophic events; such as those mediated by reactive oxygen species. Thus, sustained growth while at the same time maintaining an optimal redox state is useful for a neoplastic clone and one functional explanation for the *KRAS*-GOT1 link. Subsequent biological alterations remain currently elusive; clearly some tumors lost their GOT1 expression. The described survival differences based on the GOT1 status are not reflecting the natural course of the disease but are the result of therapeutic interference as well. However, given that neither clinical phenotype, nor proliferation fraction, or other biological features (e.g., recurrent mutations, base-exchange patterns) differed between the GOT1 subgroups, we do not have a plausible biological explanation for the strikingly different survival at this time. Thus, from a basic science perspective further exploration is warranted [21–24] and similarly, it remains to be determined whether the GOT1-status associated prognostic differences are present in other glutamine-addicted cancers as well [22, 23].

In summary, we report the GOT1 tumor tissue status as an independent prognostic biomarker in pancreatic ductal adenocarcinoma.

## MATERIALS AND METHODS

### Study Design

We performed an institutional review-board approved retrospective biomarker study to test the potential impact of GOT1 in PDAC. The study is in accord with the precepts established in the Helsinki Declaration. We explored the PDAC dataset provided by the International Cancer Genome Consortium (ICGC data portal: http://dcc.icgc.org/; last accessioned Sept., 20^th^, 2013), followed by evaluation in a separate, cohort of PDAC (ULM). These two datasets were combined into a *screening cohort*. We then assessed GOT1 protein expression in an additional, independent *validation cohort.*

### Study cohorts

The ICGC *cohort* consisted of 57 patients selected based on availability of: a) whole exome data, b) clinicopathological and follow-up data, and c) genome wide expression data (gene expression omnibus dataset GSE36924; http://www.ncbi.nlm.nih.gov/geo/; last accessioned Sept., 20^th^, 2013) [26]. The previously published ULM *cohort* is comprised of tumor resection specimens from 122 patients diagnosed between December 1997 and February 2009 [27]. *KRAS* genotyping was performed using a 5% sensitive pyrosequencing assay on a ProMarkQ24 sequencer (Qiagen, Hilden, Germany) [33]. The *validation cohort* consists of a consecutive series of primary tumor biopsy samples from 140 patients diagnosed between March 2009 and March 2013. The ULM and *validation cohort* samples consisted of routine diagnostic, formalin-fixed paraffin embedded (FFPE) material stored in a non-air-conditioned room in the basement of our institution. These samples were identified by ICD-O and keyword searches in our laboratory information system and each tumor diagnosis was evaluated by at least two board-certified pathologists. Tumor typing (ductal adenocarcinoma) and grading followed WHO criteria [20] and staging was performed according to established American Joint Committee on Cancer/TNM criteria (http://www.cancerstaging.org; last accessioned Dec., 19^th^, 2013).

### GOT1 *biomarker definitions*

At the protein level (ULM/*validation cohort*), we defined GOT1-positive (GOT1+) as cytoplasmic staining in the tumor cells that was stronger than that in the surrounding stroma; the remaining cases were considered GOT1-negative (GOT1–). We employed a commercially available goat-anti-GOT1 antibody (ab 85857; Abcam, Cambridge, MA, USA) raised against a synthetic peptide corresponding to the C-terminal amino-acids 157–167 of human GOT1 (Antigen retrieval: heat-based, citrate buffer at pH6; primary antibody at 1:100 dilution, alkaline phosphatase detection system) [34]. Specificity of staining was ascertained using hepatocytes as a positive control (either as an internal control in case of liver biopsy samples or stained in parallel as a separate positive control); stromal cells and blood vessels functioned as negative intrinsic controls and omission of the primary antibody was employed as a technical negative control. Microscopic images were captured using an Olympus BX51 or a whole-slide scanning system (.slide, both Olympus, Hamburg, Germany). At the RNA level (ICGC *cohort*), we defined GOT1-high when log2-normalized RNA probe-set expression values were above 10.3; the remaining cases were considered ‘GOT1-low’. The cutoff was determined using a stringent non-linear 4^th^ polynomic model (see Supplemental Figure 1a).

### Data collection and survival analysis

Medical records were reviewed to extract data on clinicopathologic characteristics and outcome. In the ULM and validation cohort, therapy was administered according to national guidelines [35]. The primary end point was overall survival (OS), measured from the time of initial diagnosis until the date of death. End of follow up for ULM was May 2013 and for the *validation cohort* February 2014. Patients were censored if they were lost to follow-up or if they were alive and well.

### Statistical analyses

Consisted of Spearman correlation coefficient, Fisher's exact test for dichotomous variables, *Χ^2^* test for multiple comparisons, or *t*-test for comparison of continuous variables. The Kaplan-Meier method was used to estimate overall survival. In addition to survival analysis, we modeled probability of death in the *screening cohort* (survival function including the 95% confidence intervals) and superposed and compared the event curve of the *validation cohort* (including or excluding stage IV patients). Furthermore, we used uni- as well as multivariate Cox proportional-hazards regression models to analyze survival data. Given survival times, final life status (alive or dead) and one (univariate) or more (multivariate) covariates, the regression models produce a baseline survival curve and covariate coefficient estimates with their standard errors, 95% confidence intervals, and significance levels. The covariates included in these analyses were (parenthesis provide values set to 1): sex (male), age (≥ 65), c/pT (3/4), c/pN (1), c/pM (1), Stage (III/IV), Grade (III/IV), *KRAS* (mut) and GOT1 (neg,). In a second step, we combined factors demonstrating significance in univariate assessments in our multivariate model (screening cohort: n = 175; validation cohort n = 79 patients with complete data for models). Log hazard ratios are provided with the 95% confidence intervals (CI) and statistical significance was defined as *P* < 0.05. Data analysis was conducted using Prism 6.0 (GraphPad Software Inc., San Diego, CA, USA) or online available resources (http://statpages.org/prophaz.html; last accessioned April, 3^rd^, 2014).

## SUPPLEMENTARY FIGURES AND TABLES



## Abbreviations

CI: confidence interval
FFPE: formalin-fixed
GOT1: glutamic-oxaloacetic transaminase 1 (also known as aspartate transaminase 1 or AST1)
ICGC: International Cancer Genome Consortium
AJCC: American Joint Committee on Cancer
OS: overall survival
PDAC: pancreatic ductal adenocarcinoma

## References

[1] Jemal A, Siegel R, Ward E, Hao Y, Xu J, Murray T, Thun MJ (2008). Cancer statistics. CA Cancer J Clin.

[2] Siegel R, Naishadham D, Jemal A (2013). Cancer statistics. CA Cancer J Clin.

[3] Li D, Xie K, Wolff R, Abbruzzese JL (2004). Pancreatic cancer. Lancet.

[4] Hidalgo M (2010). Pancreatic cancer. N Engl J Med.

[5] Pezzilli R, Corinaldesi R, Morselli-Labate AM (2011). Medical therapy for advanced pancreatic cancer: work in progress. JOP.

[6] Chan E, Berlin J (2014). Timing versus duration of adjuvant therapy for pancreatic cancer: all the lessons we need in life are taught to us as children. J Clin Oncol.

[7] Gorovets D, Saif MW, Huber K (2014). Novel treatment approaches for locally advanced pancreatic cancer. JOP.

[8] Huguet F, Girard N, Guerche CS, Hennequin C, Mornex F, Azria D (2009). Chemoradiotherapy in the management of locally advanced pancreatic carcinoma: a qualitative systematic review. J Clin Oncol.

[9] Yu M, Ting DT, Stott SL, Wittner BS, Ozsolak F, Paul S, Ciciliano JC, Smas ME, Winokur D, Gilman AJ, Ulman MJ, Xega K, Contino G (2012). RNA sequencing of pancreatic circulating tumour cells implicates WNT signalling in metastasis. Nature.

[10] Kleger A, Perkhofer L, Seufferlein T (2014). Smarter drugs emerging in pancreatic cancer therapy. Ann Oncol.

[11] Bhat K, Wang F, Ma Q, Li Q, Mallik S, Hsieh TC, Wu E (2012). Advances in biomarker research for pancreatic cancer. Curr Pharm Des.

[12] Costello E, Greenhalf W, Neoptolemos JP (2012). New biomarkers and targets in pancreatic cancer and their application to treatment. Nat Rev Gastroenterol Hepatol.

[13] McCleary-Wheeler AL, Lomberk GA, Weiss FU, Schneider G, Fabbri M, Poshusta TL, Dusetti NJ, Baumgart S, Iovanna JL, Ellenrieder V, Urrutia R, Fernez-Zapico ME (2012). Insights into the epigenetic mechanisms controlling pancreatic carcinogenesis. Cancer Lett.

[14] Plate JM (2012). Advances in therapeutic vaccines for pancreatic cancer. Discov Med.

[15] Roberts NJ, Klein AP (2012). Genome-wide sequencing to identify the cause of hereditary cancer syndromes: with examples from familial pancreatic cancer. Cancer Lett.

[16] Ballehaninna UK, Chamberlain RS (2013). Biomarkers for pancreatic cancer: promising new markers and options beyond CA 19-9. Tumour Biol.

[17] Garcea G, Neal CP, Pattenden CJ, Steward WP, Berry DP (2005). Molecular prognostic markers in pancreatic cancer: a systematic review. Eur J Cancer.

[18] Rousseau P (2013). How long do I have?. JAMA internal medicine.

[19] Woelk CJ (2009). How long have I got?. Can Fam Physician.

[20] Hruban RH, Boffetta P, Hiraoka N, Iacobuzio-Donahue C, Kato Y, Korn SE, Klimstra DS, Klöppel G, Maitra A, Offerhaus GJA, Pitman MB (2010). Tumours of the Pancreas. WHO Classification of Tumours of the Digestive System.

[21] Son J, Lyssiotis CA, Ying H, Wang X, Hua S, Ligorio M, Perera RM, Ferrone CR, Mullarky E, Shyh-Chang N, Kang Y, Fleming JB, Bardeesy N (2013). Glutamine supports pancreatic cancer growth through a KRAS-regulated metabolic pathway. Nature.

[22] Wise DR, Thompson CB (2010). Glutamine addiction: a new therapeutic target in cancer. Trends Biochem Sci.

[23] Wu MC, Arimura GK, Yunis AA (1978). Mechanism of sensitivity of cultured pancreatic carcinoma to asparaginase. Int J Cancer.

[24] Lyssiotis CA, Son J, Cantley LC, Kimmelman AC (2013). Pancreatic cancers rely on a novel glutamine metabolism pathway to maintain redox balance. Cell Cycle.

[25] Biankin AV, Waddell N, Kassahn KS, Gingras MC, Muthuswamy LB, Johns AL, Miller DK, Wilson PJ, Patch AM, Wu J, Chang DK, Cowley MJ, Gardiner BB (2012). Pancreatic cancer genomes reveal aberrations in axon guidance pathway genes. Nature.

[26] Perez-Mancera PA, Rust AG, van der Weyden L, Kristiansen G, Li A, Sarver AL, Silverstein KA, Grutzmann R, Aust D, Rummele P, Knosel T, Herd C, Stemple DL (2012). The deubiquitinase USP9X suppresses pancreatic ductal adenocarcinoma. Nature.

[27] Schmid SJ, Glatzel MC, Welke C, Kornmann M, Kleger A, Barth TF, Fulda S, Lennerz JK, Moller P (2013). Absence of FLICE-inhibitory protein is a novel independent prognostic marker for very short survival in pancreatic ductal adenocarcinoma. Pancreas.

[28] Tempero MA, Mokenge PM, Behrman SW, Benson AB, Casper ES, Chiorean EG, Chung V, Cohen SJ, Czito B, Feng M, Hawkins WG, Herman J, Hoffman JP (2014). NCCN Clinical Practice Guidelines in Oncology: Pancreatic Adenocarcinoma. National Comprehensive Cancer Network.

[29] Sankpal UT, Maliakal P, Bose D, Kayaleh O, Buchholz D, Basha R (2012). Expression of specificity protein transcription factors in pancreatic cancer and their association in prognosis and therapy. Curr Med Chem.

[30] Kastle M, Woschee E, Grune T (2012). Histone deacetylase 6 (HDAC6) plays a crucial role in p38MAPK-dependent induction of heme oxygenase-1 (HO-1) in response to proteasome inhibition. Free Radic Biol Med.

[31] Smith AK, White DB, Arnold RM (2013). Uncertainty--the other side of prognosis. N Engl J Med.

[32] Hayes DF, Ethier S, Lippman ME (2006). New guidelines for reporting of tumor marker studies in breast cancer research and treatment: REMARK. Breast Cancer Res Treat.

[33] Nagel PD, Feld FM, Weissinger SE, Stenzinger A, Moller P, Lennerz JK (2014). Absence of BRAF and KRAS hotspot mutations in primary mediastinal B-cell lymphoma. Leuk Lymphoma.

[34] Weissinger SE, Keil P, Silvers DN, Klaus BM, Moller P, Horst BA, Lennerz JK (2013). A diagnostic algorithm to distinguish desmoplastic from spindle cell melanoma. Mod Pathol.

[35] Seufferlein T, Adler G (2009). Med Klin (Munich).

