# Supplementary material for: GOT1/AST1 expression status as a prognostic biomarker in pancreatic ductal adenocarcinoma
Source: Oncotarget. 2015 Feb 19;6(6):4516–26. doi: 10.18632/oncotarget.2799 (PMC4414208; doi:10.18632/oncotarget.2799)
Supplement: Supplementary file 1 [file oncotarget-06-4516-s001.pdf]

SUPPLEMENTARY FIGURES AND TABLES

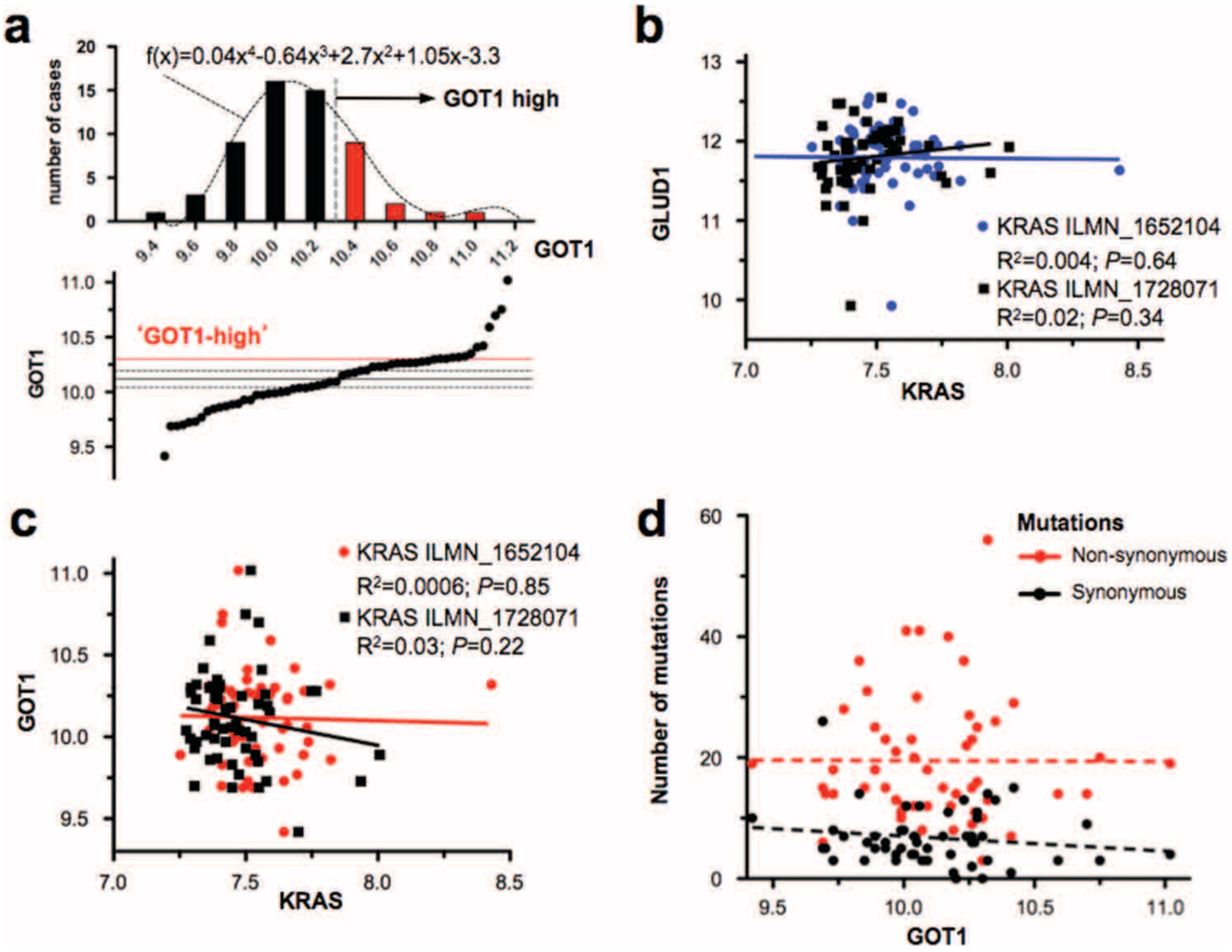

Supplementary Figure 1: Definition of GOT1-high vs. low tumors and correlation of *KRAS*- and GLUD1/GOT1 expression levels in the *ICGC cohort*. (a) lower part. GOT1-overexpression cutoff (red at 10.3 = ~2 standard deviations above the mean) displayed over individual values sorted in ascending order. Mean, black line; 95% confidence interval, hatched lines. **(a) upper part.** Frequency distribution of GOT1 expression levels (binned in 0.2-steps). The stringent cutoff-value of 10.3 (see lower part) used to define GOT1- overexpressing tumors maps beyond the maximum of a non-linear regression curve fit model. The formula of a centered 4th order polynomic model is provided.**(b, c)** Regression plots demonstrating no correlation between KRAS expression levels and GLUD1 (b) or GOT1 (c); R2-values from Pearson correlation. **(d)** Correlation of GOT1 expression levels with number of synonymous (black) and non-synonymous (red) mutations.

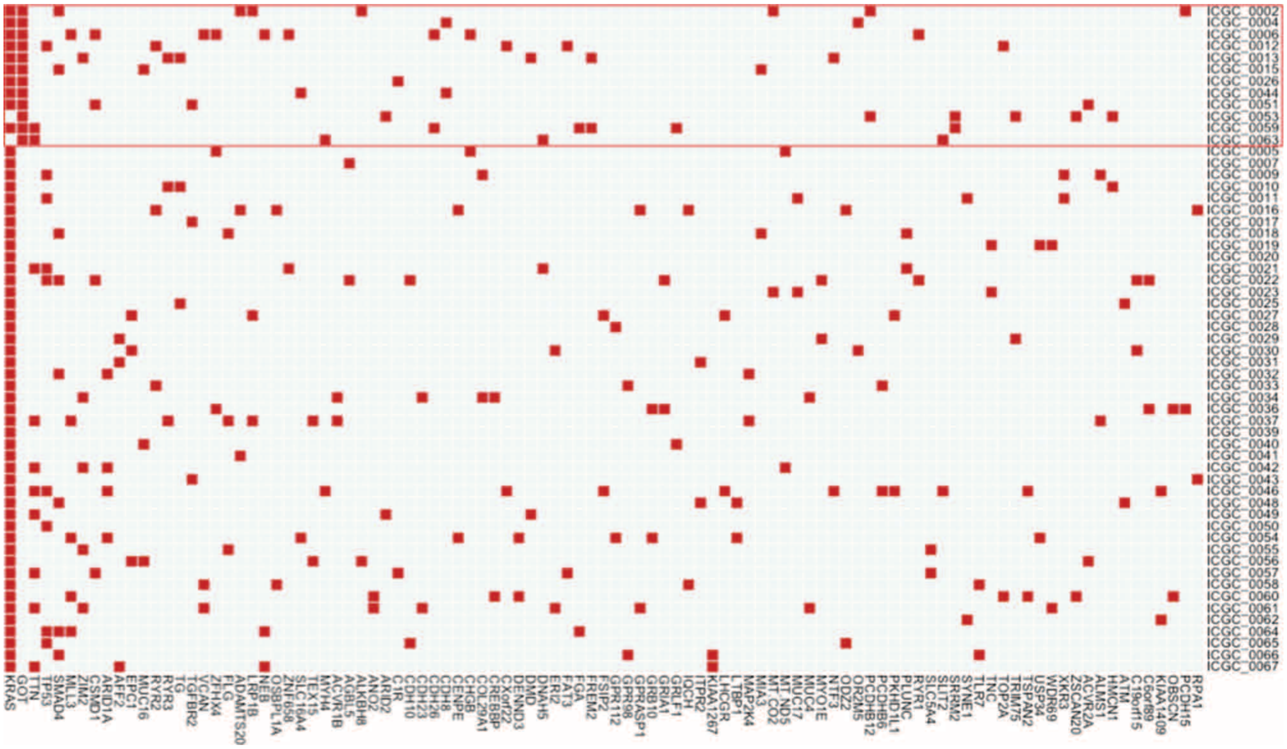

Supplementary Figure 2: Mapping of recurrent genomic mutations according to the GOT1 expression status. Genes (columns) are sorted (from left to right) by total number of recurrent mutations in the ICGC cohort (sample ID's rows). Red outline indicates GOT1- overexpressing tumors.

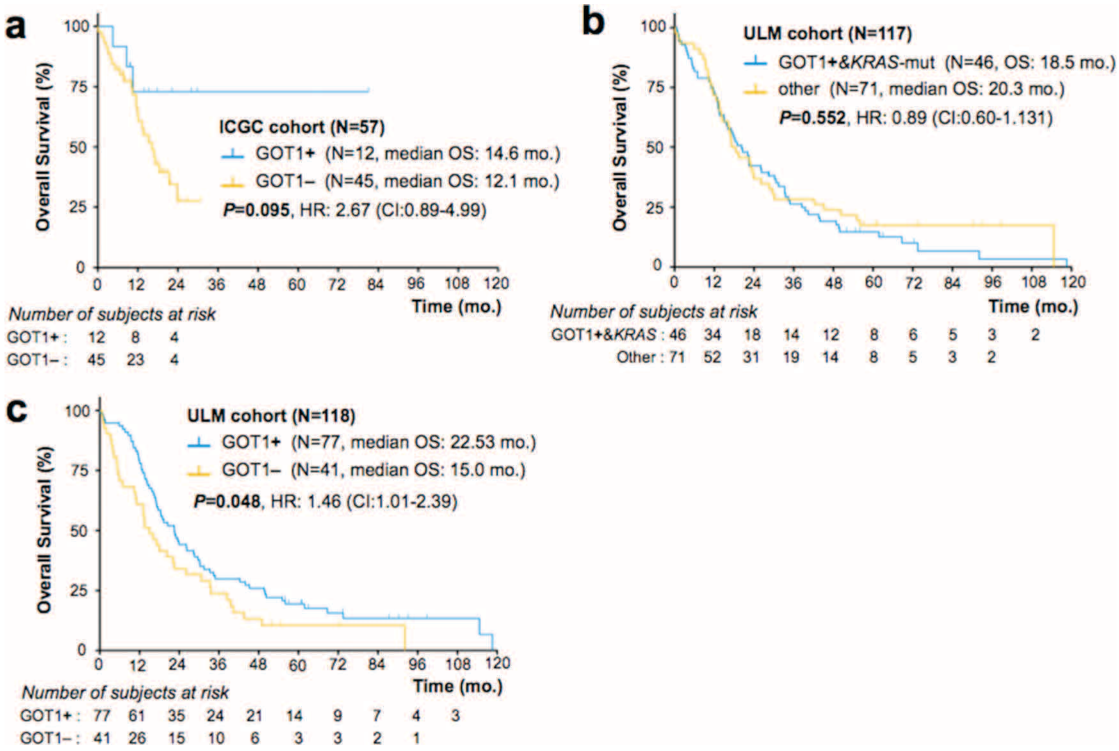

Supplementary Figure 3: Overall survival in the ICGC and ULM cohorts. (a) Kaplan-Meier estimates of overall survival in the *ICGC cohort* according to GOT1-status. **(b)** Kaplan-Meier estimates of overall survival in the *ULM cohort* comparing patients with a *KRAS*-mutation *and* GOT1-positive tumors to the remaining patients ('other'). **c.,** Kaplan-Meier estimates of overall survival in the *ULM cohort* according to GOT1-status

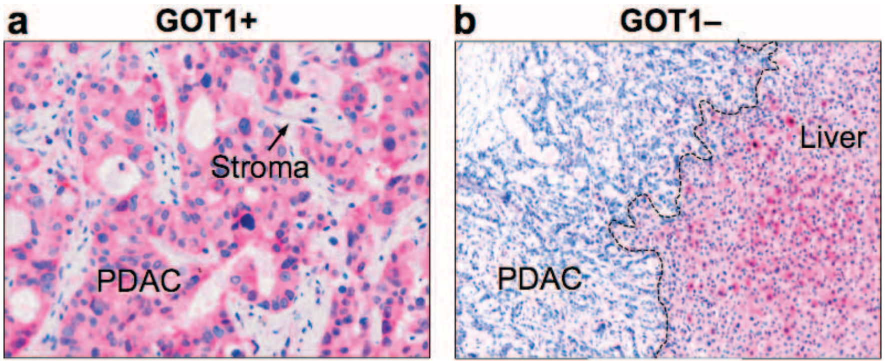

Supplementary Figure 4: GOT1 Immunohistochemistry. Immunohistochemical staining pattern of GOT1 in a positive (left, **(a)** and a negative case (right, **(b)**. GOT1+ is defined as cytoplasmic immunoreactivity (red, PDAC) that is more intense than that in the stroma (arrow). Note, liver cells show immunoreactivity and can act as an internal staining control, when present.

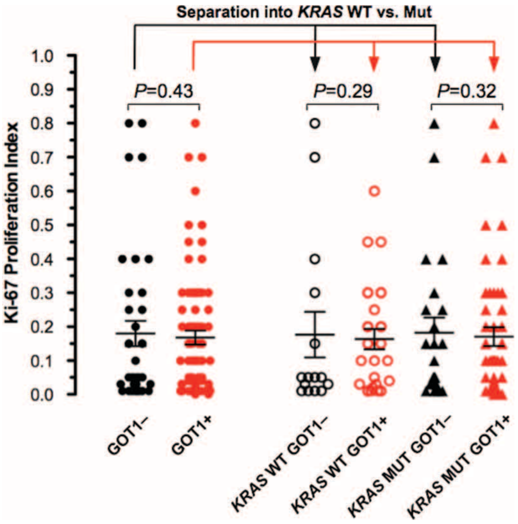

Supplementary Figure 5: Comparison of proliferation index based on GOT1 and combined *KRAS*/GOT1-status. Proliferation index, as determined by Ki-67 labeling, did not differ between the various subgroups. *P* values derived from student's t-test.

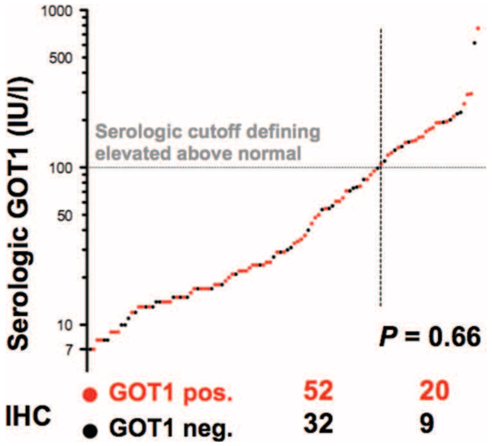

Supplementary Figure 6: Correlation of serologic GOT1 levels with immunohistochemical GOT1 status in pancreatic tumors. GOT1 immunopositivity was present in cases without abnormally high GOT1 serum levels (52/84 = 62%) as well as in cases with abnormally high GOT1 serum levels (20/29 = 69%). Thus, there is no correlation between GOT1-expression status in the tumor and the serologic GOT1 value at time of diagnosis. Abbreviation: IHC, immunohistochemistry; IU/l, international units per liter; *P* value derived from chi-square test.

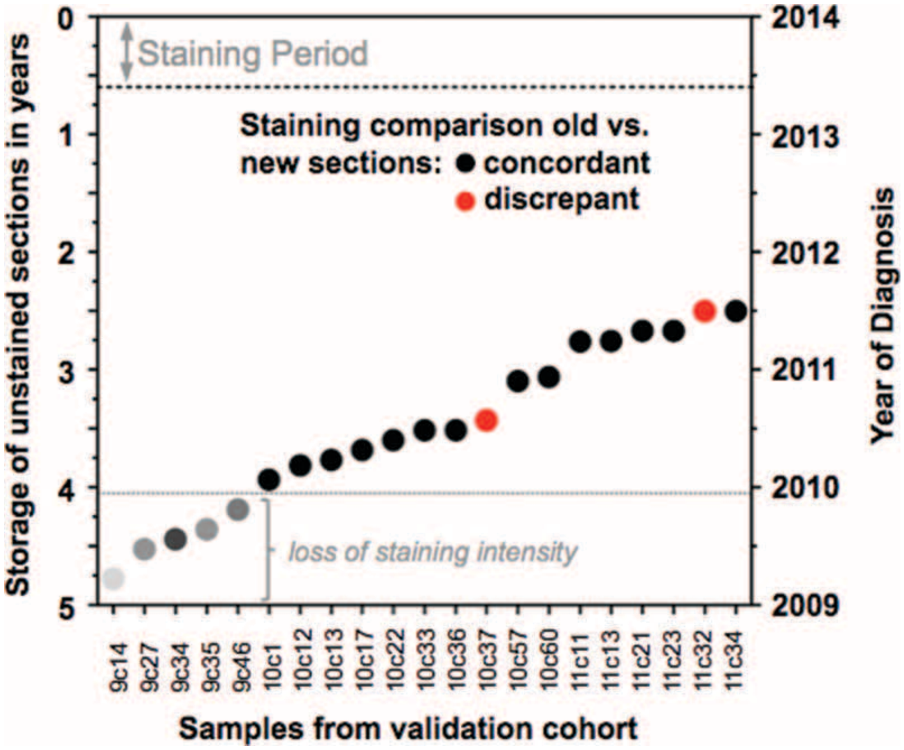

Supplementary Figure 7: Robustness of GOT1 immunohistochemistry. As part of our institutional policy, unstained tissue sections at the time of original diagnosis are stored along with stained sections in a non-air-conditioned room in the basement of our institution. To test robustness of immunohistochemical GOT1 detection in tissue sections, we used these 'aged' unstained sections and correlated the detected staining pattern with freshly cut and stained tissue sections from the FFPE-block stored in the same location. Cases are sorted by storage-time and based on staining quality defined as concordant staining of tumor and internal positive and negative control (i.e., liver and stroma, respectively). GOT1 can be reliably detected in precut sections stored up to 4 years and there is a ~90% concordance to newly cut and stained tissue sections, indicating that GOT1 detection is reliable and stable over time.

Supplementary Table 1: Clinicopathological features by GOT1 status – comparison between the ICGC and ULM cohort

	ICGC *N* = 57		*P*	ULM *N* = 122		*P*	*P* GOT1+ ICGC vs. Ulm	*P* GOT1- ICGC vs. Ulm
**Characteristic**	**GOT1+ *N* = 12**	**GOT1- *N* = 45**		**GOT1+ *N* = 77**	**GOT1- *N* = 41**			
**Sex, *n* (%)**								
male	5 (42%)	29 (64%)	0.19	41 (53%)	28 (68%)	0.12	0.52	0.82
female	7 (58%)	16 (36%)		36 (47%)	13 (32%)			
**Age**								
Median	68.5	64.0		65.7	69.6			
Range	49–81	34–87		40–82	43–83			
Age <65 n (%)	4 (33%)	24 (52%)	0.33	36 (47%)	15 (37%)	0.33	0.54	0.14
Age ≥65 n (%)	8 (66%)	21 (48%)		41 (53%)	26 (63%)			
**Site, *n* (%)**								
Head	11 (92%)	36 (80%)	0.67	68 (88%)	33 (83%)	0.41	1.00	0.79
Body/Tail	1 (8%)	9 (20%)		9 (12%)	7 (17%)			
**Tumor Size, *n* (%)**								
T1/2	4 (33%)	5 (11%)	0.07	10 (13%)	2 (5%)	0.21	0.09	0.44
T3/4	8 (66%)	40 (89%)		67 (87%)	39 (95%)			
**Nodal Metastasis, *n* (%)**								
N0	2 (17%)	9 (20%)	1.00	22 (29%)	14 (34%)	0.54	0.50	0.22
N1	10 (83%)	35 (80%)		55 (71%)	27 (66%)			
**Metastasis, *n* (%)**								
M0	11 (92%)	42 (93%)	1.00	72 (94%)	37 (90%)	0.72	1.00	0.70
M1	1 (8%)	3 (7%)		5 (6%)	4 (10%)			
**Stage Grouping, *n* (%)**								
I/II	11 (92%)	42 (93%)	1.00	69 (90%)	34 (83%)	0.39	1.00	0.18
III/IV	1 (8%)	3 (7%)		8 (10%)	7 (17%)			
**Differentiation *n* (%)**								
G1/2	10 (83%)	24 (52%)	0.10	55 (72%)	27 (66%)	0.54	0.50	0.28
G3/4	2 (17%)	21 (48%)		22 (28%)	14 (33%)			

Abbreviations: GOT1, Glutamic Oxalacetic Transaminase 1; stage grouping according to AJCC

Supplementary Table 2: Jaundice and serologic phenotype screening

Characteristic	available	GOT1+ *N* = 77	GOT1- *N* = 41	*P*
**Clinical *n* (%)**				
Jaundice, J1	64	43 (56%)	21 (53%)	0.845
Jaundice, J0	53	34 (44%)	19 (47%)	
Stenting, yes	50	33 (43%)	17 (43%)	1.000
Stenting, no	67	44 (57%)	23 (57%)	
**Laboratory**				
Bilirubin ≥ 2mg/dl	51	33 (43%)	18 (43%)	1.000
Bilirubin < 2mg/dl	67	44 (57%)	23 (57%)	
sGOT1 ≥ 100 IU/l	34	25 (32%)	9 (22%)	0.288
sGOT1 < 100 IU/l	84	52 (68%)	32 (78%)	
AP ≥ 120 IU/l	68	46 (61%)	23 (56%)	0.696
AP < 120 IU/l	48	30 (39%)	18 (44%)	

Note: the tumor (i.e., tissue-based) GOT1-status status does not correlate with jaundice or serologic findings. Abbreviations: AP, alkaline phosphatase; IU, international units; l, liter; sGOT1, serum glutamic oxaloacetate transaminase 1 (also known as alanine aminotransferase 1, AST1).
